# Ultrasound shear wave elastography of breast lesions: correlation of anisotropy with clinical and histopathological findings

**DOI:** 10.1186/s40644-018-0144-x

**Published:** 2018-04-05

**Authors:** Ya-ling Chen, Yi Gao, Cai Chang, Fen Wang, Wei Zeng, Jia-jian Chen

**Affiliations:** 1 Department of Ultrasound, Fudan University Shanghai Cancer Center, No. 270 Dong-An Road, Shanghai, 200032 China; 2 Department of Oncology, Shanghai Medical College, Fudan University, No. 270 Dong-An Road, Shanghai, 200032 China; 3 Department of Breast Surgery, Fudan University Shanghai Cancer Center, No. 270 Dong-An Road, Shanghai, 200032 China

**Keywords:** Breast lesion, Ultrasound, Elasticity, Shear wave elastography, Anisotropy, BI-RADS

## Abstract

**Background:**

Ultrasound shear-wave elastography (SWE) may increase specificity of breast lesion assessment with ultrasound, but elasticity measurements may change with transducer orientation, defined as anisotropy. In this study, we aimed to observe the anisotropy of SWE of breast lesions, and its correlation with clinical and histopathological findings.

**Methods:**

This retrospective study was approved by institutional review board. From June 2014 to June 2015, a total of 276 women (mean age, 48.75 ± 12.12 years) with 276 breast lesions (174 malignant, 102 benign) were enrolled for conventional ultrasound and SWE before surgical excision. Elasticity modulus in the longest diameter and orthogonal diameter were recorded, including maximum elasticity (Emax), mean elasticity (Emean), standard deviation (Esd) and ratio between mean elasticity of lesion and normal fatty tissue (Eratio). Anisotropy coefficients including anisotropic difference (AD) and anisotropy factors (AF) were calculated, and correlations with malignancy, tumor size, palpability, movability, lesion location and histopathology were analyzed.

**Results:**

The average Emax, Emean, Esd and Eratio of the longest diameter were significantly higher than orthogonal diameter (*P* < 0.05). AUCs of ADs and AFs were inferior to quantitative parameters (*P* < 0.001), with AUCs of AFs superior to ADs (*P* < 0.001). ADs showed no significant correlation with malignancy, palpability, movability, distance from nipple and skin, and histopathological patterns. ADmean was significantly higher in inner half than outer half of the breast (*P* = 0.034). Higher AFs were significantly correlated with larger lesion size (*P* = 0.042), palpability (*P* < 0.05), shorter distance from nipple and skin (*P* < 0.05) and higher suspicion for malignancy (*P* < 0.001). AFs were significantly higher in IDC than DCIS (*P* < 0.05), higher in Grade II/III than Grade I IDC (*P* < 0.001), and correlated with ER/PR(+) (*P* < 0.05).

**Conclusions:**

AF of SWE was an indicator for malignancy and more aggressive breast cancer.

## Background

Ultrasound (US) is a useful routine tool in screening and differentiation of benign and malignant breast lesions [1, 2]. The Breast Imaging-Reporting and Data System (BI-RADS) lexicon of American College of Radiology (ACR) has been widely applied in clinical practice [3]. In recent years, breast ultrasonic elastography has become a new promising technique obtaining more accurate characterization of breast lesions [4, 5]. Among the currently used elastography technique, shear wave elastography (SWE) induces shear waves which propagate transversely in the tissue, and has been confirmed as a quantitative stiffness measurement technique of high reproducibility and less operator dependency, compared to external mechanical compression based strain elastography [4, 6]. Previous studies demonstrated that combination of conventional US with SWE features significantly improved specificity of breast mass assessment without loss of sensitivity [7–11], and thus could reduce unnecessary biopsies of low-suspicion BI-RADS category 4A masses.

When performing SWE examination, the imaging planes used in reported studies of SWE have varied. In some studies, SWE images were acquired in a single transducer orientation for each mass [7, 9]. However, in other studies, two orthogonal planes were obtained routinely, either radial/antiradial planes or transverse/longitudinal planes [10, 12], and diagnostic performance was improved by combining conventional ultrasound with two-view SWE (two orthogonal planes) compared with combining with single-view SWE (single transducer orientation) [12].

Anisotropy is an orientation-dependent property that exists in fiber-rich tissues, which implies different properties in different directions. In terms of ultrasound elastograpy, anisotropy could be defined as different imaging features with the change of orientation of the transducer, resulting in different measurements of elasticity when assessing along different axes. Recently, Zhou et al. has demonstrated the anisotropy of elasticity of normal breast glandular and fatty tissue by comparing measurements of radial and antiradial planes [13]. Previous studies observed anisotropy in solid breast lesions [14], and Skerl et al. discovered anisotropy in SWE as an indicator of malignancy [15]. Nevertheless, in the aforementioned study, the anisotropy factor (AF) was calculated with Emean, which was defined as mean elasticity of the stiffest area using a region of interest size (ROI) of 2 mm, rather than the measurements of the whole lesions. Besides, anisotropy of other quantitative parameters such as Emax, Esd and Eratio has not been analyzed yet [15].

The aim of this study is to observe the anisotropy of each SWE quantitative parameter of breast lesions between two orthogonal planes, and its correlation with clinical and histopathological findings in Chinese patients.

## Materials and methods

### Patients

A retrospective analysis of 284 consecutive women with 284 breast lesions detected by palpation and/or imaging was performed from June 2014 to June 2015. All participants were inpatients from department of Breast Surgery of our center, and underwent conventional US and 2-dimensional (2D) SWE before surgical excision. Eight patients with large masses (over 4 cm) which couldn’t be covered by SWE colour overlay were excluded. Finally, 276 women (mean age, 48.75 ± 12.12 years; age range, 21–84 years) with 276 breast lesions constituted the study cohort.

### Image acquisition

Conventional US and 2D SWE were performed using the Aixplorer® US system (SuperSonic Imagine, Aix-en-Provence, France) with a SL15–4 multifrequency linear-array transducer by one of three radiologists with 5–20 years’ experience in breast imaging (Y.L.C., Y.G. and F.W.). Prior to this clinical trial, all participating investigators had performed over 4000 breast US examinations in two years, and had practiced breast SWE on over 200 cases for the last 6 months. We firstly used the default preset of breast, with center frequency at “GEN”, dynamic range at 70 dB, tissue tuner 1480 m/s. We decreased the center frequency to “PEN” if lesions were deeply located, while increased to “RES” with superficial location. The clockwise location, distance from the nipple and the skin were recorded.

SWE was carried out at default scale -- 180 kPa. Three acquisitions through the longest diameter of the lesion (View A) and another three acquisitions through the orthogonal diameter plane (View B) were obtained and saved for analysis.

### Image evaluation

Before SWE examination, independent and blinded review of conventional US images was performed by two investigators (C.C. and W.Z.) with 20 years of experience in breast US, and classified into appropriate categories according to ACR BI-RADS US [3].

Quantitative SWE features were measured on each SWE images of View A and View B using the quantification tool built in Aixplorer® US system. By using a circular ROI covering as much as the entire lesion and any immediately adjacent stiff areas on the SWE images, we measured maximal elasticity (Emax), mean elasticity (Emean), standard deviation of elasticity (Esd) of the whole lesion. The ratio between the mean elasticity of the lesion and normal fatty tissue (Eratio) was calculated with the same circular ROI of 2 mm of diameter placed on the stiffest portion of the lesion (or its immediately adjacent tissue) and normal fatty tissue respectively. Average values for each parameter of three acquisitions in both View A and View B were calculated.

### Anisotropy

To evaluate the anisotropic properties of SWE of breast lesions, anisotropy coefficients were calculated to quantify the differences in elasticity between View A and View B through the equations below [15]. The anisotropic difference (AD) for Emax, Emean, Esd and Eratio was calculated as

$$ \mathrm{ADmax}={\mathrm{Emax}}_{\mathrm{View}\ \mathrm{A}}\hbox{--} {\mathrm{Emax}}_{\mathrm{View}\ \mathrm{B}},\mathrm{ADmean}={\mathrm{Emean}}_{\mathrm{View}\ \mathrm{A}}\hbox{--} {\mathrm{Emean}}_{\mathrm{View}\ \mathrm{B}} $$

$$ \mathrm{ADsd}={\mathrm{Esd}}_{\mathrm{View}\ \mathrm{A}}\hbox{--} {\mathrm{Esd}}_{\mathrm{View}\ \mathrm{B}},\mathrm{ADratio}={\mathrm{Eratio}}_{\mathrm{View}\ \mathrm{A}}\hbox{--} {\mathrm{Eratio}}_{\mathrm{View}\ \mathrm{B}} $$

The anisotropy factor (AF) was calculated as the square of AD to evaluate the general anisotropy of the lesion independent on the stiffer plane:

$$ \mathrm{AF}={\mathrm{AD}}^2 $$

### Clinical findings

Clinical data of each patient was recorded, such as palpability, movability and location of the lesions. When recording the location, we divided the breast into four quadrants, including upper inner quadrant, upper outer quadrant, lower inner quadrant and lower outer quadrant, and assessed the location according to the center of the lesion. According to the nipple level, the breast was divided into upper half (upper inner quadrant and upper outer quadrant) and lower half (lower inner quadrant and lower outer quadrant). Upper inner quadrant and lower inner quadrant constituted the inner half, while upper outer quadrant and lower outer quadrant constituted the outer half.

### Histopathologic examination

All the lesions enrolled underwent surgical excision, and histopathological outcome was used as the Gold Standard, which was made by a pathologist with 20 years of experience in breast pathology who was blinded to the US results.

### Statistical analysis

Statistical analyses were performed by Y.L.C and J.J.C using SPSS, version 19.0 (SPSS, Chicago, IL, USA). Receiver operating characteristic (ROC) curves were analyzed using MedCalc for Windows, version 15.6 (MedCalc Software, Mariakerke, Belgium). The area under ROC curves (AUC) for conventional US, quantitative parameters of SWE and anisotropy coefficients were calculated for diagnostic performance analysis. The optimal cutoff values were determined with the Youden index. Comparison of AUC was performed using the method proposed by DeLong et al. [16]. Anisotropy coefficients were compared between benign and malignant lesions, using the Kruskal-Wallis test. Nonparametric tests for trend were used for analysis across ordered groups. Spearman correlation coefficient (ρ) was used for correlation analysis. A *P* < 0.05 was considered to indicate a statistically significant difference.

## Results

The histopathological results of the 276 lesions were shown in Table 1, among which 174 (63.0%) were malignant, and 102 (37.0%) were benign. The average of maximal diameter at conventional US was 15.65 ± 5.57 mm (range, 6–31 mm; median 14.76 mm), with malignant lesions significantly larger than benign lesions (19.70 ± 6.02 mm vs. 15.28 ± 5.24 mm, *P* < 0.001). Except for 32 (11.6%) lesions detected by imaging, the rest 244 (88.4%) were palpable, among which 104 lesions were movable.

**Table 1:** Pathologic Diagnosis of 276 Breast Lesions

Pathologic Diagnosis	No. of Lesions	Percent
Malignant Lesions	174	
Invasive ductal Carcinoma	156	89.7
Invasive lobular Carcinoma	4	2.3
Ductal carcinoma in situ	13	7.5
Mucinous adenocarcinoma	1	0.6
Benign Lesions	102	
Fibroadenoma	59	57.8
Adenosis	19	18.6
Intraductal papilloma	19	18.6
Benign phyllodes tumor	1	1.0
Mastitis	4	3.9

### Quantitative elasticity of two orthogonal planes

**Fig. 1:**
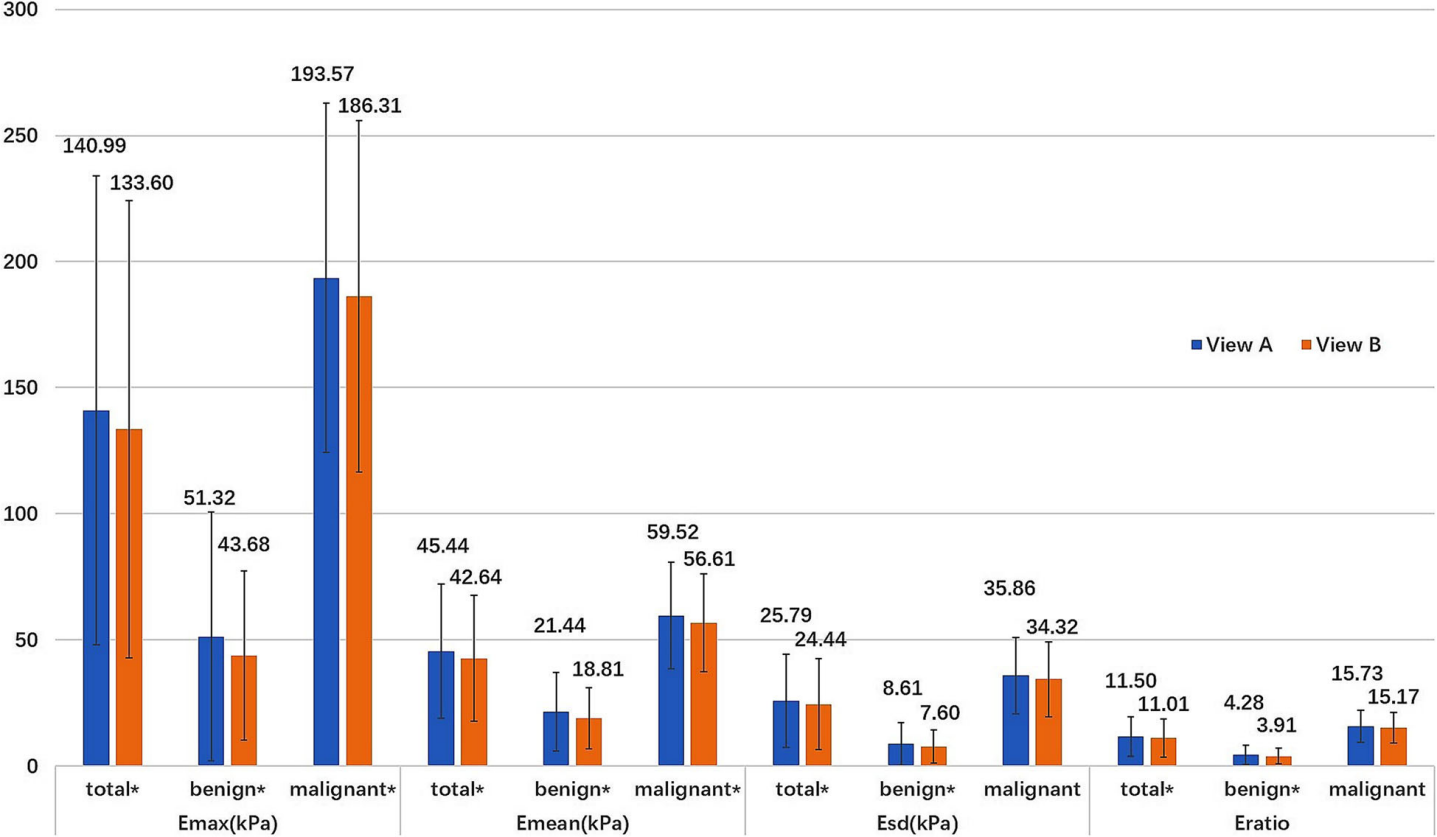
Histogram of quantitative elasticity of 276 breast lesions in the longest diameter plane and the orthogonal diameter plane. * Quantitative elasticity was significantly higher in the longest diameter plane (View A) than orthogonal diameter plane (View B): *P* < 0.05

**Fig. 2:**
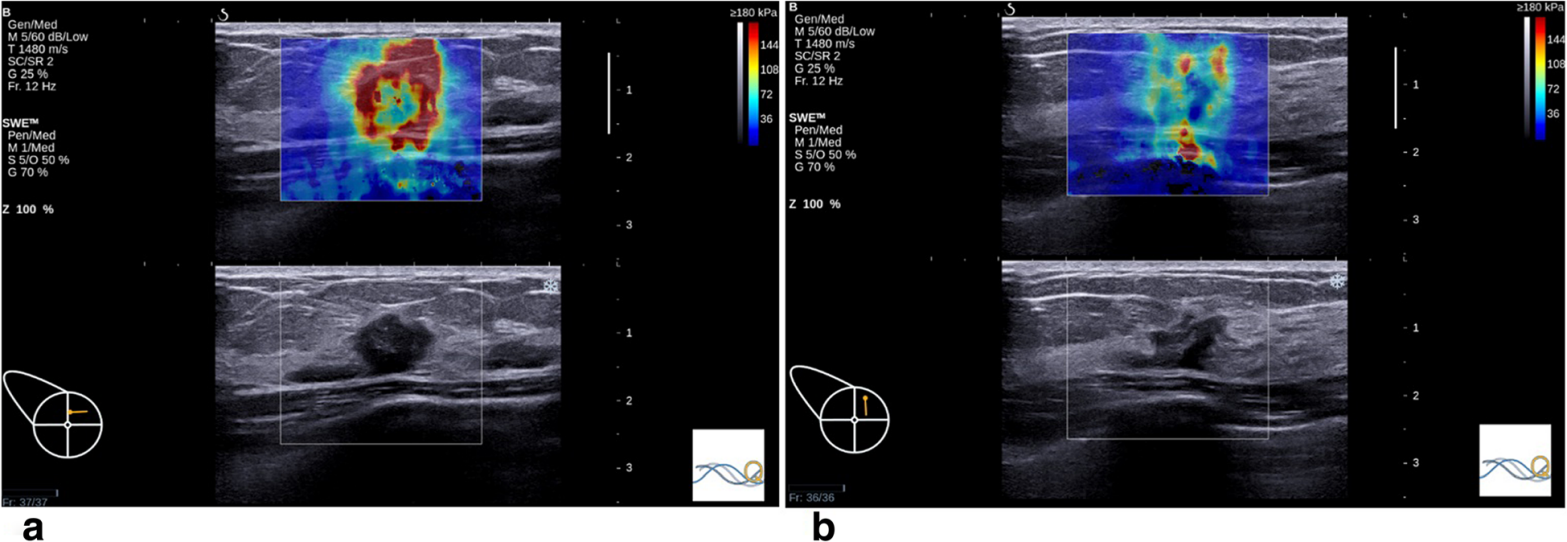
The longest diameter plane (View A) showed higher elasticity than the orthogonal diameter plane (View B) in a malignant lesion. A mass in the upper-inner quadrant of right breast of a 45-year-old woman was histopathologically confirmed as invasive ductal carcinoma (Grade II). **a** In View A, Emax, Emean and Esd were 300 kPa, 145.4 kPa and 61.9 kPa, respectively. **b** In View B, Emax, Emean and Esd were 164.2 kPa, 70.9 kPa and 25.9 kPa, respectively

Both by considering the total lesions together and the benign group alone, the average Emax, Emean, Esd and Eratio were significantly higher in View A than View B (*P* < 0.05). In the malignant group, Emax and Emean were significantly higher in View A than View B (*P* < 0.05), without significant difference for Esd and Eratio Figs. 1, 2.

All the quantitative parameters (Emax, Emean, Esd and Eratio) in View A and View B were significantly higher in malignant group than benign group (*P* < 0.001) Fig. 1.

### Anisotropy of quantitative parameters of SWE

**Fig. 3:**
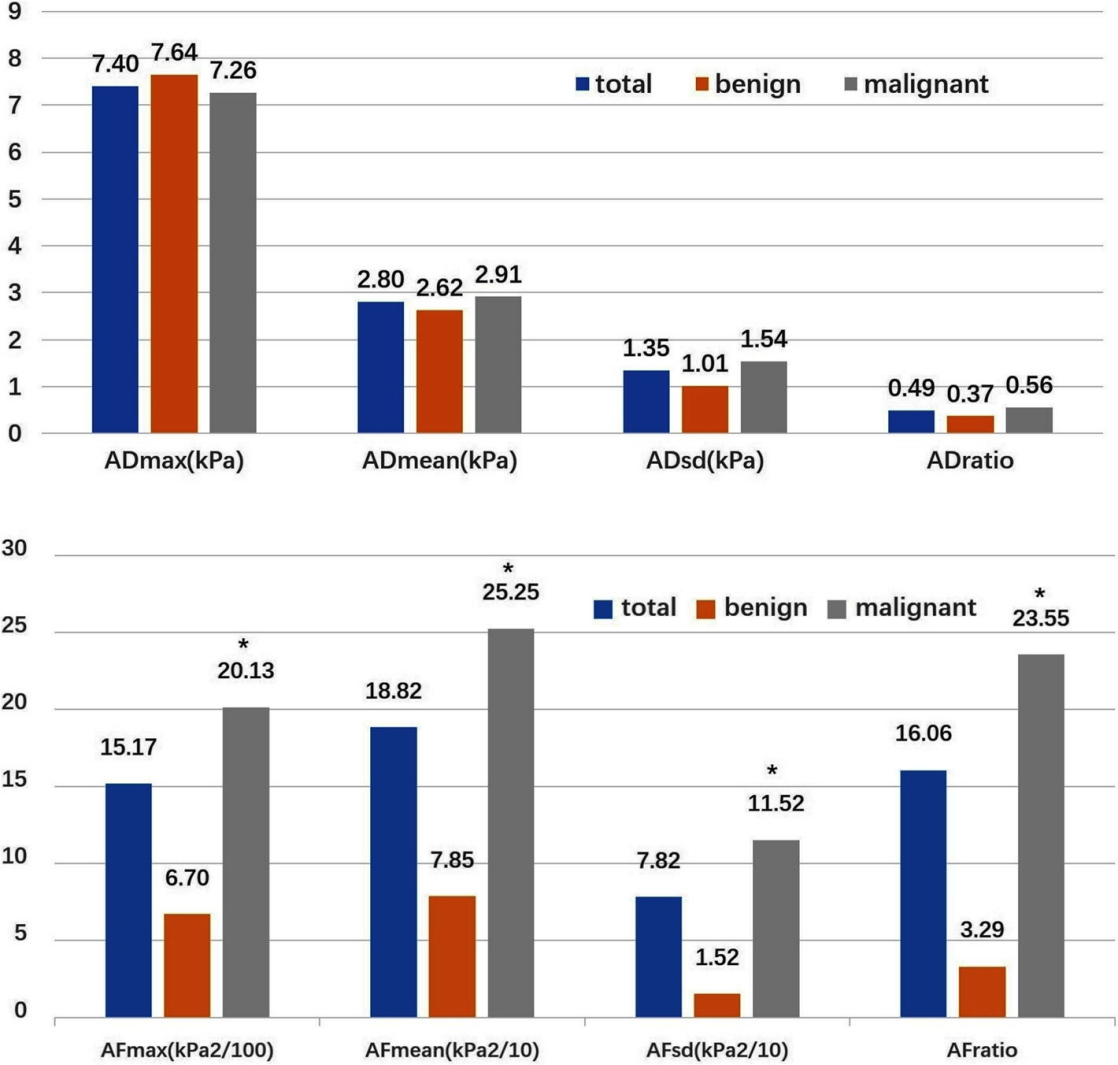
Anisotropy factor (AF) was significantly higher in malignant lesions than in benign lesions (*P* < 0.001), while anisotropic difference (AD) did not show significant difference. * malignant vs. benign: *P* < 0.05

We calculated the AD and AF of Emax, Emean, Esd and Eratio between two orthogonal planes. ADs showed positive correlation with quantitative parameters of in View A (*P* < 0.001) while negative correlation with View B (*P* < 0.01). AFs showed positive correlation with quantitative parameters (Emax, Emean, Esd and Eratio) (*P* < 0.001). ADs didn’t show significant difference between malignant and benign lesions. However, AFs were significantly higher in malignant lesions than in benign lesions (*P* < 0.001) Fig. 3.

### Diagnostic performance of anisotropy coefficient

**Fig. 4:**
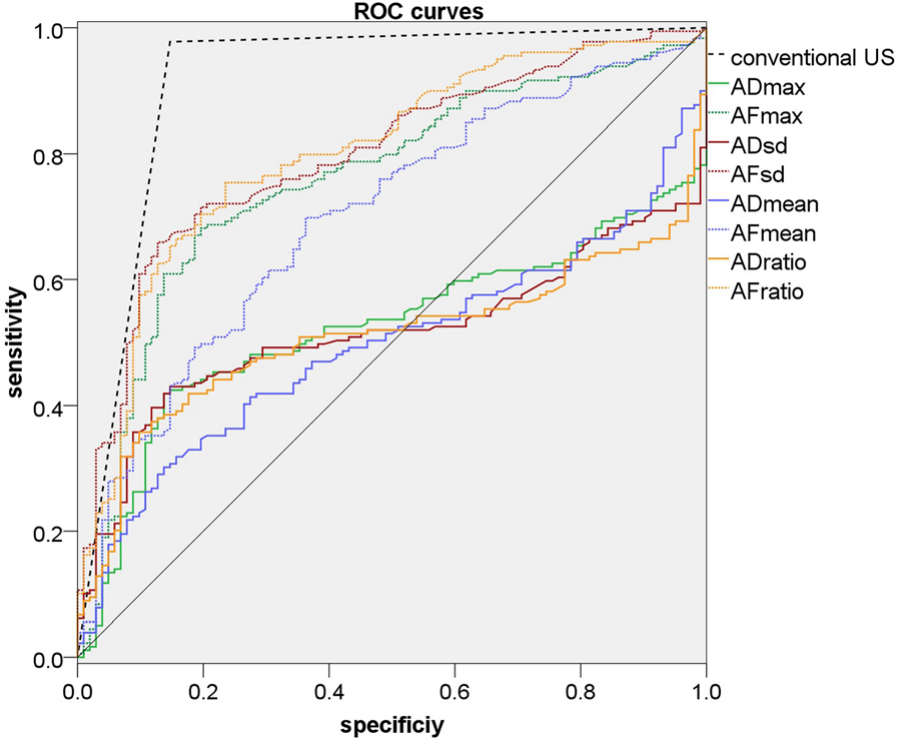
ROC curves of anisotropic difference (ADs) and anisotropy factor (AFs) compared to conventional US assessment. The diagnostic performance of ADs and AFs was inferior to that of conventional US, while diagnostic performance of AFs was superior to that of ADs

AUC of conventional US according to BI-RADS was 0.918, with cutoff value between BI-RADS 4A and 4B. AUCs of ADs and AFs were inferior to AUCs of quantitative parameters (Emax: 0.940, Emean: 0.921, Esd: 0.944, Eratio: 0.940) and conventional US (*P* < 0.001), while AUCs of AFs (AFmax: 0.760, AFmean: 0.702, AFsd: 0.802, AFratio: 0.804) were superior to ADs (ADmax: 0.525, ADmean: 0.501, ADsd: 0.516, ADratio: 0.512) (*P* < 0.001), with optimal cutoff value higher than 159.52kPa^2^ (AFmax), 21.44kPa^2^ (AFmean), 10.89kPa^2^ (AFsd) and 1.35 (AFratio) Fig. 4.

**Fig. 5:**
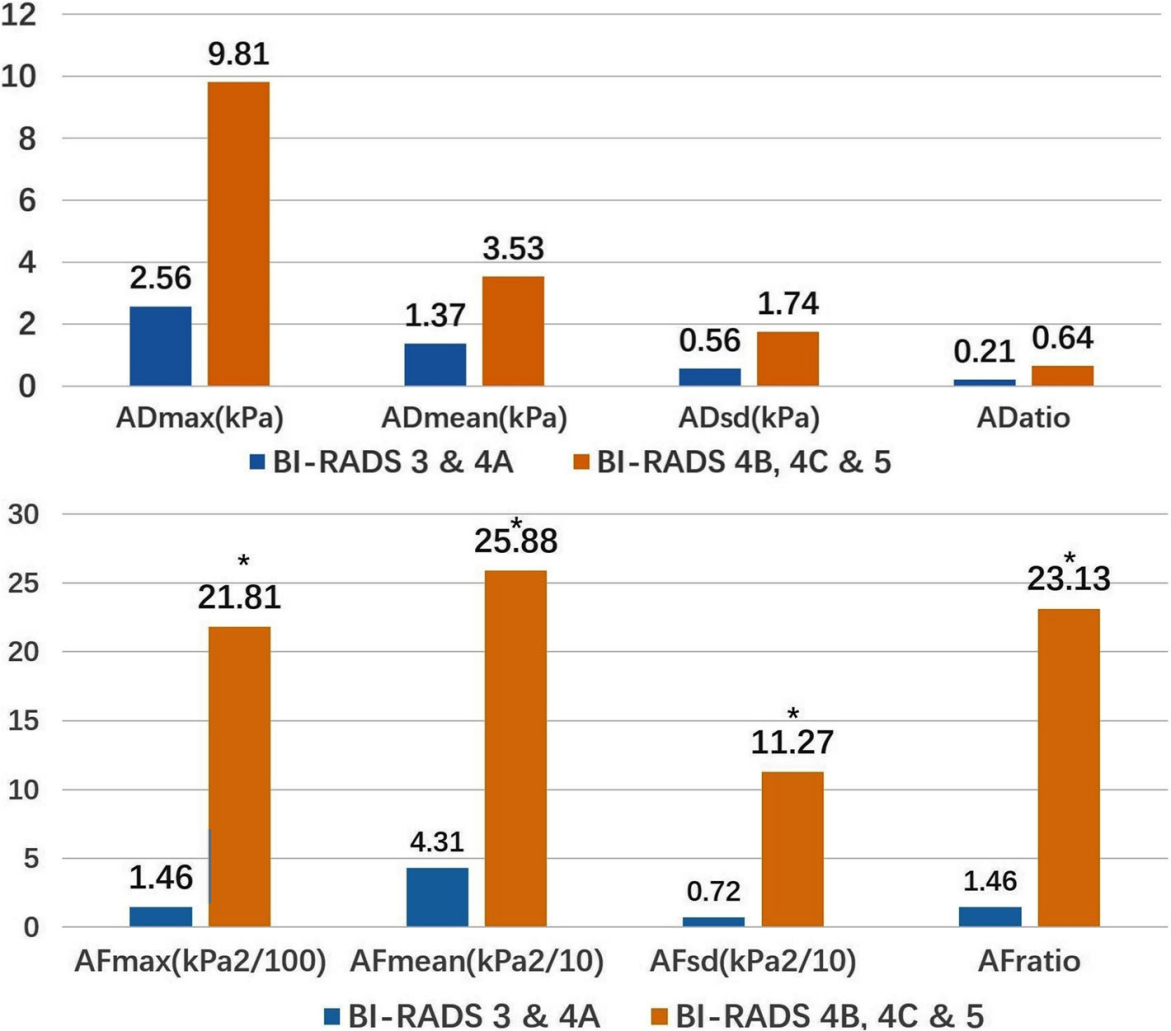
Correlation between anisotropy and malignancy. Anisotropy factor (AF) was significantly higher in high-suspicious group (BI-RADS 4B, 4C & 5) than in low-suspicious group (BI-RADS 3 & 4A) (*P* < 0.001), while anisotropic difference (AD) showed no significant difference. * high-suspicious group vs. low-suspicious group: *P* < 0.05

All the SWE quantitative parameters (Emax, Emean, Esd and Eratio) were significantly higher in high-suspicious group (BI-RADS 4B, 4C & 5) than in low-suspicious group (BI-RADS 3 & 4A) (*P* < 0.001). ADs showed no significant difference between two groups (*P* > 0.05), while AFs were significantly higher in high-suspicious group than in low-suspicious group (*P* < 0.001) Fig. 5.

### Correlation of anisotropy coefficients with lesion size

The total lesions were divided into large lesions group (≥ 15 mm) and small lesions group (< 15 mm) according to the cutoff value calculated by ROC analysis in our study cohort (≥ 15 mm). A cut-off threshold of 15 mm was used also because it was between the median (14.76 mm) and the mean (15.45 mm) of the lesion size, and therefore, gave groups of similar numbers.

**Fig. 6:**
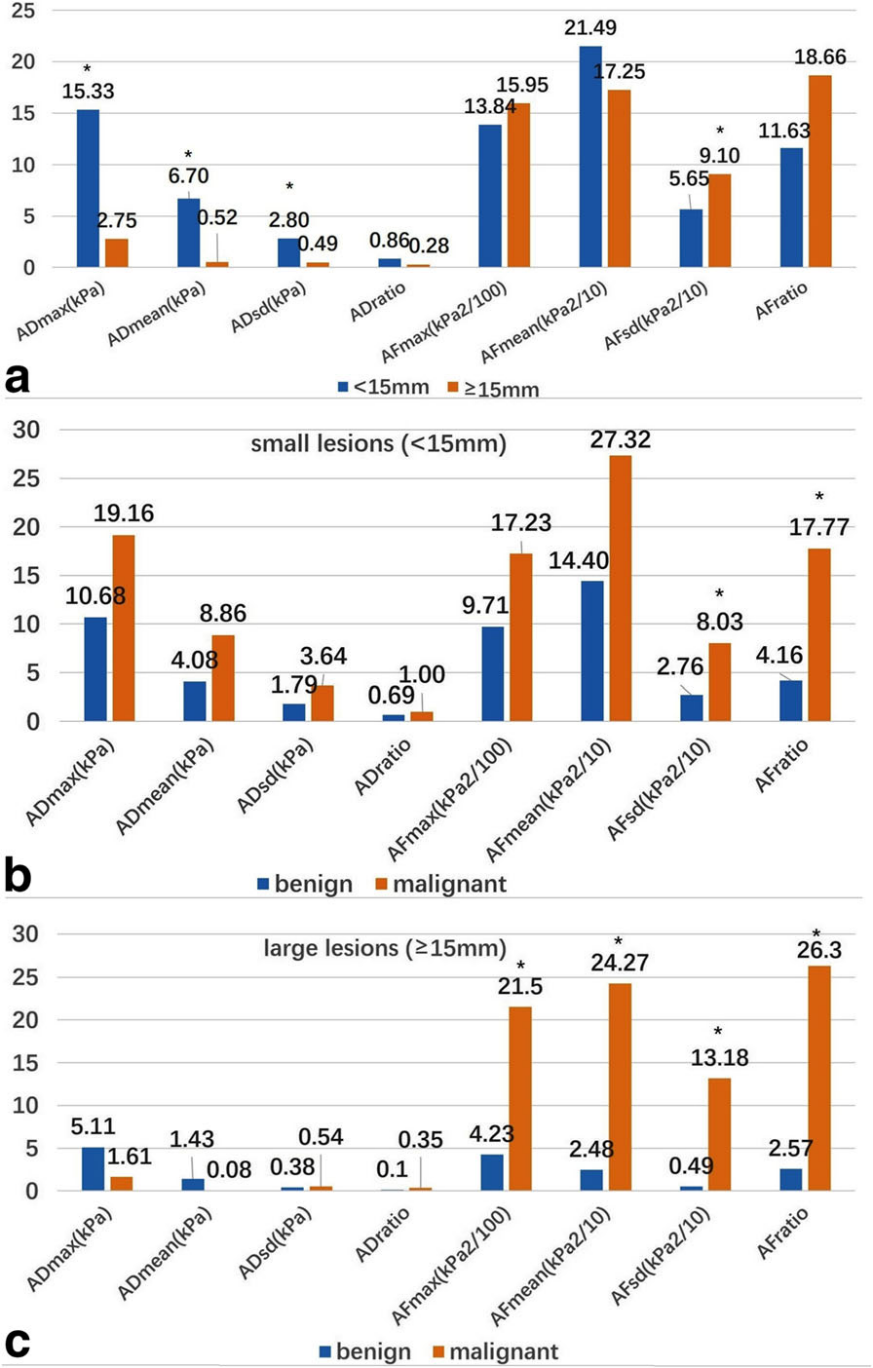
Correlation between anisotropy and lesion size. ADmax, ADmean and ADsd were significantly higher in small lesions, while AFsd was significantly higher in large lesions (**a**). AD did not show significant difference between malignant and benign lesions, either in small lesions (**b**) and large lesions (**c**). AF was significantly higher in malignant lesions both in small lesions (**b**) and large lesions (**c**)

Quantitative parameters Emax, Esd and Eratio were significantly higher in large lesions than small lesions (*P* < 0.001), while Emean did not show significant difference. ADmax, ADmean and ADsd were significantly higher in small lesions than large lesions (*P* < 0.05), while AFsd was significantly higher in large lesions (*P* = 0.042). ADs did not show significant difference between malignant and benign group, either in large lesions or small lesions. AFs were significantly higher in malignant lesions than benign lesions both in large lesions (AFmax, AFmean, AFsd and AFratio: *P* < 0.001) and in small lesions (AFsd: *P* = 0.020; AFratio: *P* = 0.005) Fig. 6.

### Correlation of anisotropy coefficients with clinical findings

All the quantitative parameters (Emax, Emean, Esd and Eratio) were significantly higher in palpable lesions than impalpable lesions (*P* < 0.001), and significantly higher in immovable lesions than movable lesions (*P* < 0.001).

ADs did not show significant correlation with palpability and movability. AFsd and AFratio were significantly higher in palpable lesions than impalpable lesions (AFsd: *P* = 0.009; AFratio: *P* < 0.001), while AFmax, AFmean showed no significant difference between two groups. In palpable group, AFs were significantly higher in immovable lesions than movable ones (AFmax: *P* < 0.001; AFmean: *P* = 0.006; AFsd: *P* < 0.001; AFratio: *P* = 0.002) Table 2.

**Table 2:** Correlation of anisotropy factor (AF) with Clinical Findings

Clinical Findings	AFmax (kPa^2^/100)	AFmean (kPa^2^/10)	AFsd (kPa^2^/10)	AFratio
Palpable (*n* = 244)	15.71 ± 28.53	19.66 ± 43.56	8.39 ± 16.29^*^	17.54 ± 37.46^*^
Impalpable (*n* = 32)	11.00 ± 32.46	12.39 ± 20.31	3.52 ± 8.27	4.78 ± 8.13
Movable (*n* = 104)	8.27 ± 21.90	13.82 ± 37.69	4.94 ± 13.06	10.52 ± 28.49
Immovable (*n* = 140)	21.30 ± 31.45^#^	25.43 ± 50.92^#^	11.43 ± 18.76^#^	23.12 ± 42.93^#^

*AFsd and AFratio were significantly higher in palpable lesions than in impalpable ones (*P* = 0.009 and *P* < 0.001; respectively)

^#^AFmax, AFmean, AFsd and AFratio were significantly higher in immovable lesions than in movable ones (*P* < 0.001, *P* = 0.006, *P* < 0.001 and *P* = 0.002; respectively)

### Correlation of anisotropy coefficients with distance of lesions from the nipple

By analyzing the total lesions together, negative correlation was found between AFmean and distance of lesions from the nipple (*ρ* = − 0.124, *P* = 0.039).

In the benign group, all the quantitative parameters (Emax, Emean, Esd and Eratio) and AFs except AFmean showed significantly negative correlation with the distance from the nipple (*P* < 0.05) Table 3.

**Table 3:** Correlation of anisotropy coefficient with distance from the nipple and depth of lesion

Anisotropy Coefficients	Distance from nipple (Spearman’s ρ)			Depth of lesions (Spearman’s ρ)		
	Total	Benign	Malignant	Total	Benign	Malignant
ADmax	−0.006	−0.101	0.046	−0.082	− 0.094	− 0.061
ADmean	− 0.047	− 0.082	− 0.031	−0.019	− 0.083	−0.006
ADsd	−0.011	−0.111	0.032	−0.069	− 0.085	−0.045
ADratio	0.026	−0.193	0.112	−0.026	−0.015	− 0.004
AFmax	−0.005	− 0.199 ^*^	0.063	−0.125 ^#^	− 0.202 ^#^	− 0.018
AFmean	−0.124 ^*^	− 0.187	−0.131	− 0.120 ^#^	− 0.089	−0.097
AFsd	−0.010	−0.216 ^*^	− 0.003	−0.098	− 0.021	−0.087
AFratio	0.013	−0.195 ^*^	0.076	−0.104	− 0.102	−0.051

*correlation of AF with the distance from the nipple: Total lesions: AFmean *P* = 0.039. Benign group: AFmax *P* = 0.045; AFsd *P* = 0.029; AFratio *P* = 0.049

^**#**^correlation of AFs with the depth of lesions: Total lesions: AFmax *P* = 0.039; AFmean *P* = 0.046. Benign group: AFmax *P* = 0.042

### Correlation of anisotropy coefficients with the depth of lesions

By analyzing the total lesions, negative correlation with the depth of lesions was found in all the quantitative parameters (Emax, Emean, Esd and Eratio) (*P* < 0.001) and also in AFmax and AFmean (*P* < 0.05).

Nevertheless, negative correlation with the depth of lesions was only found in AFmax in the benign group (ρ = − 0.202, *P* = 0.042), and in quantitative parameters Emean (ρ = − 0.172, *P* = 0.023) and Eratio (ρ = − 0.217, *P* = 0.004) in the malignant group Table 3.

### Correlation of anisotropy coefficients with quadrant location of lesions

Analyzing the total lesions, none of the quantitative parameters (Emax, Emean, Esd and Eratio) nor anisotropy coefficients (ADs and AFs) showed significant correlation with quadrant location or upper/lower half location. Nevertheless, all the quantitative parameters (Emax, Emean, Esd and Eratio) were significantly higher in inner half than outer half (*P* < 0.001), so did the coefficient ADmean (*P* = 0.034). In the benign group, ADmean and AFmean were also significantly higher in inner half than outer half (*P* < 0.05) Table 4.

**Table 4:** Correlation of anisotropy coefficient with quadrant location

Anisotropy Coefficients	Quadrant Location					
	Total		Benign		Malignant	
	Inner half	Outer half	Inner half	Outer half	Inner half	Outer half
ADmax (kPa)	12.89 ± 35.95	5.60 ± 38.96	15.92 ± 31.42	6.10 ± 23.35	11.96 ± 37.47	5.25 ± 47.05
ADmean (kPa)	5.96 ± 14.32 ^*^	1.77 ± 13.03	8.15 ± 13.57 ^*^	1.60 ± 6.83	5.29 ± 14.61	1.89 ± 16.05
ADsd (kPa)	2.35 ± 8.39	1.02 ± 8.87	2.11 ± 5.94	0.81 ± 3.24	2.42 ± 9.06	1.16 ± 11.28
ADratio	0.13 ± 3.77	0.61 ± 4.05	0.32 ± 2.53	0.38 ± 1.63	0.07 ± 4.10	0.77 ± 5.12
AFmax (kPa^2^/100)	14.40 ± 23.45	15.42 ± 30.63	11.70 ± 27.43	5.76 ± 24.38	15.20 ± 22.32	22.32 ± 32.78
AFmean (kPa^2^/10)	23.77 ± 49.40	17.20 ± 38.68	23.97 ± 50.67 ^*^	4.87 ± 12.54	23.72 ± 49.41	25.90 ± 47.58
AFsd (kPa^2^/10)	7.49 ± 11.88	7.93 ± 16.71	3.75 ± 6.95	1.10 ± 4.21	8.64 ± 12.86	12.75 ± 20.21
AFratio	14.05 ± 23.30	16.72 ± 38.79	6.10 ± 12.04	2.77 ± 9.64	16.50 ± 25.39	26.56 ± 47.68

*Inner half vs. Outer half:

Total lesions: ADmean *P* = 0.034

Benign group: ADmean *P* = 0.004; AFmean *P* = 0.003

### Correlation of anisotropy coefficients with histopathology

All the quantitative parameters (Emax, Emean, Esd and Eratio) were significantly higher in invasive ductal carcinoma (IDC) lesions than ductal carcinoma in situ (DCIS) lesions (*P* < 0.001).

ADs did not show significant correlation with different tumor types, estrogen receptor (ER)/progesterone receptor (PR), HER2 and Ki-67 expression, and lymph node metastasis (*P* > 0.05).

AFs were significantly higher in IDC lesions than DCIS lesions (AFmax, AFsd, AFratio: *P* < 0.01). AFratio was significantly lower in Grade I IDC than Grade II and Grade III IDC (*P* < 0.001) Table 5.

**Table 5:** Correlation of anisotropy coefficient with histological grades in IDC lesions

	Grade I	Grade II	Grade III
ADmax (kPa)	−12.01 ± 22.33	13.46 ± 44.15	4.20 ± 49.42
AFmax (kPa^2^/100)	5.72 ± 13.27	21.03 ± 30.09	24.28 ± 33.24
ADmean (kPa)	−1.99 ± 5.64	4.49 ± 17.40	2.11 ± 15.30
AFmean (kPa^2^/10)	3.13 ± 3.51	31.88 ± 59.04	23.55 ± 42.15
ADsd (kPa)	−3.16 ± 7.46	2.94 ± 11.23	1.23 ± 11.06
AFsd (kPa^2^/10)	5.77 ± 8.17	13.30 ± 19.15	12.23 ± 19.84
ADratio	−0.57 ± 0.91	0.69 ± 4.69	0.69 ± 5.37
AFratio	1.05 ± 1.32	22.13 ± 38.09 ^*^	28.91 ± 48.85 ^**#**^

*AFratio: Grade I vs. Grade II, *P* < 0.001

^#^AFratio: Grade I vs. Grade III, *P* < 0.001

Some AFs were significantly higher in ER/PR positive lesions than ER/PR negative lesions [ER(+) vs. ER(−): AFsd 13.02 ± 20.28 vs. 7.33 ± 10.96 kPa^2^/10, *P* = 0.019; AFmean 29.22 ± 53.32 vs. 14.36 ± 26.72 kPa^2^/10, *P* = 0.016; PR(+) vs. PR(−): AFsd 13.59 ± 20.85 vs. 7.28 ± 11.05 kPa^2^/10, *P* = 0.01], without significant correlation with HER2, Ki-67 expression and lymph node metastasis.

## Discussion

Anisotropy is the property of being directionally dependent, which exists in biological tissues rich in fibers. As the glandular and fatty tissue organized along the ducts leading radially to the nipple, breast tissue is structurally anisotropic with radial orientation in the whole breast [17, 18]. The mechanical anisotropy created by highly aligned collagen fibers facilitates elongation and branching [19]. Recently, anisotropy of elasticity has been demonstrated in normal breast glandular and fatty tissue [13]. Owing to the propagation of shear wave that was roughly parallel to the direction of fibers of Cooper’s ligaments and ducts in the radial plane, shear wave velocity in radial plane was significantly higher than anti-radial plane in both glandular tissue and fatty tissue [13]. A previous study has demonstrated the existence of anisotropy of Emean in breast lesions, with a 2 mm ROI focused on the stiffest area of the lesion rather than the measurements of the whole lesions, without analyzing anisotropy of other SWE quantitative parameters such as Emax, Esd and Eratio [15]. The objective of our study was to investigate the anisotropy of all the quantitative parameters, with large ROI covering as much as the lesion.

In the study by Skerl et al., about half breast lesions were stiffer in radial planes and the other half stiffer in anti-radial planes [15]. Differently in our study, quantitative elasticity of breast lesions was significantly higher in longest diameter plane than orthogonal diameter plane, indicating that anisotropy did exist in elasticity of breast lesions. The different results might due to the different planes chosen for anisotropy analysis between Skerl’s study and ours. When assessing breast lesions in conventional ultrasound imaging, the longest diameter and its orthogonal plane were adopted for measurement, as a widely accepted method, rather than always measuring radial/anti-radial planes or anatomically sagittal/axial planes [3]. Because in clinical practice, breast tumors were not always oriented horizontally or vertically but sometimes obliquely within the image. As tumor cells at the tumor boundary contract and align collagen fibers with the assistance of proteolytic cleavage, and then invade along aligned collagen structure to expand the tumor and later metastasize [20]. Previous study demonstrated that there was an excellent correlation between the mean tumor stiffness value and the maximum diameter (*r* = 0.94, *P* < 0.0001) [21]. The elasticity, represented as Young’s modulus E, is positively correlated with the square of propagating speed of shear wave. Therefore, we hypothesized that shear wave propagated faster along the maximum diameter, which could explain the significantly higher elasticity in the longest diameter of the lesions, and was in agreement with that proposed by Skerl et al. [15].

Previous study by Skerl et al. demonstrated that in lesions with higher Esd value (≥7 kPa), AFs calculated by radial and anti-radial planes showed no significant difference between malignant and benign lesions, while AFs calculated by two orthogonal planes unrelated to radial orientation (sagittal/axial planes) were significantly higher in malignant lesions than benign lesions [15]. In other words, anisotropy factor calculated by two orthogonal planes unrelated to radial orientation was more predictable for malignancy than that calculated by radial/anti-radial planes for more heterogeneous lesions. In our study, the lesions enrolled were more heterogeneous according to the statistics (Esd ≥ 7 kPa) (longest diameter plane: 25.56 ± 18.49 kPa; orthogonal diameter plane: 24.32 ± 17.93 kPa), and AFs calculated by two orthogonal planes unrelated to radial orientation were significantly higher in malignant lesions than benign lesions, both in small lesions and large lesions, confirming the predictable value for malignancy.

The study by Skerl et al. calculated AF with the ROI on the stiffest 2 mm of the lesion [15], while in our study AF was calculated with elasticity of the whole lesion instead, which could provide more complete information about the elasticity and anisotropy. As mentioned above, the lesions enrolled in our study were more heterogeneous, therefore analyzing the stiffest portion of the lesion alone might lose elastic information of rest part of the lesion. In our study, AUC of AFmax was 0.760, higher than 0.67 reported by Skerl et al. [15], with lower threshold of AFmax (159 kPa^2^ vs 200 kPa^2^), indicating higher sensitivity. We also found that AFratio yielded the highest AUC (0.804) among all AF parameters, indicating anisotropy of Eratio predictable for malignancy.

This study was the first attempt to our knowledge to fully analyze anisotropy of each quantitative parameter. In previous studies, correlation between quantitative elasticity and histopathological results has been demonstrated [22–26]. Emean of IDC was significantly associated with palpable abnormality, histologic grade, and lymphovascular invasion [22], lymph node involvement and lymphovascular invasion was associated with significantly higher Emean, Emax, and Eratio [23], and higher histologic grade was significantly correlated with higher Emax [24, 25]. According to our results, AF was significantly higher in IDC than DCIS, and AFratio of Grade II and Grade III IDC was significantly higher than Grade I IDC lesions, indicating AF as an effective predictor of histological severity of breast cancer. Previous studies demonstrated that ER (−), PR (−), p53 (+), Ki-67 (−) and high nuclear grade were associated with a significantly higher Eratio (*P* < 0.05) [25]. Nevertheless in our study, AF was higher in ER (+) and PR (+) lesions, while no significant correlation with HER2, Ki-67 and lymphatic metastasis. The correlation between AF and immunohistochemical factors requires future study.

Correlation between anisotropy and lesion location was analyzed for the first time. Some of the anisotropy factors were higher in lesions located near the nipple and the skin. In other words, lesions located near the nipple and the skin tended to be more anisotropic. That might because compression artifacts more frequently occur near the skin, and the fact that mammary ducts were more convergent near the nipple. Therefore, it may have explained the result in our study that lesions in inner half of the breast tended to be stiffer and more anisotropic, since the breast tissue of inner half is usually thinner than outer half so that lesions located at inner half are likely to be nearer the skin. It reminded us to take anisotropy into account when characterizing lesions near the nipple and skin. When analyzing correlation of anisotropy with palpability, we found that palpable lesions were more anisotropic than impalpable lesions. It might due to the fact that palpable lesions usually tended to be larger or near the skin, and lesions of large size and shallow depth were more anisotropic.

Owing to the existence of anisotropy, it is important to change the transducer orientation to fully assess the lesion when performing SWE. The influence of lesion location should be considered when characterizing breast lesions with the aid of anisotropy.

There were several limitations to our study. First, the two orthogonal planes we compared were longest diameter and orthogonal diameter planes, and therefore uncertain to cover the stiffest portion of the lesions. Second, large lesions which could not be covered by SWE color overlay were excluded in our study. Since large lesions were demonstrated to be more anisotropic, the exclusion of large lesions may cause selection bias. Third, it was a retrospective study, the patients enrolled were scheduled for surgical excision, and the low-suspicious BI-RADS 3 &4A lesions only constituted 32.3% of the lesions. Since high-suspicious group was more anisotropic, statistical results may be affected by the selection bias. Fourth, the small number DCIS cases [7.5% (13/174)] among the malignant group could have statistically influenced the results when comparing anisotropy between IDC and DCIS, and further study of large sample would be needed for validation.

## Conclusions

Our study indicated that AF was superior to AD in predicting malignancy. Higher anisotropy was associated with higher suspicion for malignancy and more aggressive breast cancer. Taking anisotropy into account when performing breast SWE may help to characterize breast lesions and predict prognosis of cancer.

## Abbreviations

2D: Two-dimensional
ACR: American College of Radiology
AD: Anisotropic difference
AF: Anisotropy factors
AUC: Areas under ROC curves.
BI-RADS: Breast Imaging Reporting and Data System
Emax: Maximal elasticity
Emean: Mean elasticity
Eratio: Ratio between the mean elasticity in the lesion and the fatty tissue
Esd: Standard deviation of elasticity
ROC: Receiver operating characteristic
SWE: Shear wave elastography
US: Ultrasound
